# Genome-Wide Identification and Expression Analysis of *NPF* Genes in Cucumber (*Cucumis sativus* L.)

**DOI:** 10.3390/plants12061252

**Published:** 2023-03-09

**Authors:** Mengying Zhang, Wenyan Zhang, Zijian Zheng, Zhiping Zhang, Bing Hua, Jiexia Liu, Minmin Miao

**Affiliations:** 1 College of Horticulture and Landscape, Yangzhou University, Yangzhou 225009, China; 2 Joint International Research Laboratory of Agriculture and Agri-Product Safety of Ministry of Education of China, Yangzhou University, Yangzhou 225009, China; 3 Key Laboratory of Plant Functional Genomics of the Ministry of Education, Jiangsu Key Laboratory of Crop Genomics and Molecular Breeding, Yangzhou University, Yangzhou 225009, China

**Keywords:** cucumber, nitrogen, *NPF* genes, abiotic stress, nitrate transporter

## Abstract

The NITRATE TRANSPORTER 1/PEPTIDE TRANSPORTER family (NPF) proteins perform an essential role in regulating plant nitrate absorption and distribution and in improving plant nitrogen use efficiency. In this study, cucumber (*Cucumis sativus* L.) *NPF* genes were comprehensively analyzed at the whole genome level, and 54 *NPF* genes were found to be unevenly distributed on seven chromosomes in the cucumber genome. The phylogenetic analysis showed that these genes could be divided into eight subfamilies. We renamed all *CsNPF* genes according to the international nomenclature, based on their homology with *AtNPF* genes. By surveying the expression profiles of *CsNPF* genes in various tissues, we found that *CsNPF6.4* was specifically expressed in roots, indicating that *CsNPF6.4* may play a role in N absorption; *CsNPF6.3* was highly expressed in petioles, which may be related to NO_3_^−^ storage in petioles; and *CsNPF2.8* was highly expressed in fruits, which may promote NO_3_^−^ transport to the embryos. We further examined their expression patterns under different abiotic stress and nitrogen conditions, and found that *CsNPF7.2* and *CsNPF7.3* responded to salt, cold, and low nitrogen stress. Taken together, our study lays a foundation for further exploration of the molecular and physiological functions of cucumber nitrate transporters.

## 1. Introduction

Nitrogen (N), a life element, is involved in the synthesis of macromolecules and secondary metabolites in plants and is closely related to crop yield and quality [1]. In agricultural production, nitrogen fertilizer is applied to increase crop yields. Applying a limited amount of nitrogen fertilizer is insufficient for supplying the requirements for plant growth and development, leading to reduced yields and income. However, excessive application of nitrogen fertilizer greatly reduces the nitrogen utilization rate of plants, causes a waste of resources, and results in a series of environmental pollution problems, such as soil hardening and greenhouse gas emissions [2,3]. Consequently, how to improve plant nitrogen use efficiency and realize rational fertilization are urgent problems to be solved in agricultural production.

Nitrogen in soil is mainly found in the form of inorganic nitrogen (nitrate (NO_3_^−^) and ammonium (NH_4_^+^)) and organic nitrogen (urea, nucleotide and amino acids). Nitrate is an essential N source used by higher plants and can be taken up by plant roots from the soil through nitrate transporters (NRTs) and then translocated within the plants [4]. Generally, four protein families, including the nitrate transporter 1/peptide transporter family (NPF), nitrate transporter 2 (NRT2), chloride channel family (CLC), and slow anion channel-associated homologs (SLAC/SLAH), are involved in nitrate transport [5]. As the largest family of nitrate transporter proteins in plants, NPFs are widely present in higher plants with diverse functions and have received widespread attention. To date, the identification of the *NPF* gene family has been completed in many crops, with 53 *NPF* family members identified in the model plant *Arabidopsis thaliana* [6], 74 identified in rice (*Oryza sativa*) [7], 331 identified in wheat (*Triticum aestivum*) [8], and 79 identified in maize (*Zea mays*). In horticultural crops, 57, 44, and 77 *NPF* genes were identified in spinach (*Spinacia oleracea*) [9], pineapple (*Ananas comosus*) [10], and apple (*Malus domestica*) [11], respectively.

To date, the significant functions of *NPF* genes in nitrate utilization in Arabidopsis have been revealed. *NPF4.6/NRT1.2* and *NPF6.3/NRT1.1* were found to be involved in nitrate absorption in plant roots. Among them, *AtNPF6.3/NRT1.1* [12,13,14] is considered a dual-affinity nitrate transporter that is regulated by N status. In contrast to *AtNPF6.3*, *AtNPF4.6/AtNRT1.2* [15] only encodes a low-affinity transporter protein involved in the NO_3_^−^ absorption process. In addition to absorbing nitrate, some transport proteins in plant roots are responsible for the excretion of nitrate. To maintain the nitrate balance in plants, *AtNPF2.7/NAXT1* [16] is responsible for NO_3_^−^ efflux from roots in acidic environments. In addition, many *NPF* genes are involved in the transport and distribution of nitrogen in plant vegetative organs. *AtNPF2.3* [17] mediates the transport of nitrate from the roots to aboveground parts under salt stress conditions. *AtNPF2.9/NRT1.9* [18] is responsible for nitrate loading in the root phloem and regulates nitrogen transport between the roots and shoots. *AtNPF5.11*, *AtNPF5.12*, and *AtNPF5.16* [19] can transport nitrate stored in vacuoles to the cytoplasm under low nitrogen conditions and participate in the regulation of nitrate distribution between the roots and shoots. In addition to the absorption of NO_3_^−^, *AtNPF6.3* is involved in the long-distance transport of NO_3_^−^ from the roots to aboveground parts. *AtNPF7.3/NRT1.5* [20,21] is involved in the loading of nitrate in the xylem, while *AtNPF7.2/NRT1.8* [22] is responsible for NO_3_^−^ retrieval from the xylem in roots and is involved in the response to adversity stress. *AtNPF6.2/NRT1.4* [23,24] is involved in NO_3_^−^ storage in petioles, which also affects the root/stem distribution of potassium. *AtNPF1.1/NRT1.12* and *AtNPF1.2/NRT1.11* [25] are required for distributing nitrate from mature tissues to young tissues, while *AtNPF2.13/NRT1.7* [26,27] is expressed in the phloem of old leaves and is required for N remobilization in old leaves. The transport and distribution of nitrogen in reproductive organs are also the responsibility of specific *NPF* genes. *AtNPF2.12/NRT1.6* [28] is responsible for nitrate transport to developing embryos and participates in the nitrogen cycle in plants under low nitrogen stress. *AtNPF5.5* [29] is involved in nitrogen accumulation in embryos.

In addition to their involvement in nitrogen utilization, *NPF* genes with multiple functions in other physiological processes are also being identified. *AtNPF4.4/NRT1.13* [30] monitoring of the nitrate level in the interior near the xylem can adjust the branch structure and flowering time. Moreover, NPF is a key transporter of many other substrates. *AtNPF4.6* transports abscisic acid (ABA), to regulate the stomatal aperture, and *AtNPF6.3/NRT1.1* is involved in nitrate-induced auxin transport and regulation of root morphology. *AtNPF2.4* [31] is involved in the long-distance transport of Cl^−^ in plants and mediates the storage of Cl^−^ in the root xylem of Arabidopsis during salt stress. *AtNPF2.5* [32] mediates the Cl^−^ efflux from the root. *AtNPF7.3* [20] may be responsible for the loading of proton-coupled potassium into the xylem. Furthermore, NPF is involved in adversity stress. It has been shown that *AtNPF6.3* is generally associated with plant tolerance to various adverse environmental conditions, such as Cd^2+^, H^+^, Na^+^, Pb^2+^, Zn^2+^, and drought stress [33]. These reports provided further evidence of the importance of NPF for plant growth and development.

The cucumber (*Cucumis sativus* L.) is one of the most important vegetables worldwide. Cucumber is a shallow root crop with a relatively poor ability to absorb fertilizers and water, and its growth and development strongly depend on the nitrogen supply. Therefore, the study of NPF transporter proteins is important for improving the nitrogen utilization efficiency in the cucumber. However, only a few studies have focused on the cucumber *NPF* gene family. Previously, 13 cucumber *NPF* genes were identified and subjected to a detailed expression analysis [34]. Examining the effects of 24-epibrassinolide (EBR) on cucumber seedlings under suboptimal root zone temperature (RZT) showed that suboptimal RZT significantly downregulated the expression of *CsNRT1* genes in cucumber leaves, but an exogenous EBR application significantly induced the expression of these genes under suboptimal RZT [35]. Studies investigating the expression patterns of major *NRT1* family members in cucumber roots showed that excessive NO_3_^−^ stress increased the transcript level of *NRT1.5A* but repressed the expression of *NRT1.1* and *NRT1.8*, and Si promoted the expression of *NRT1.8* [36]. After verifying the accumulation and localization of mRNA using qPCR and RNA in situ hybridization, it was found that the *CsNPF7.2* gene was induced by short-term nitrogen deficiency, which may be related to the development of plant vascular bundles under nitrogen stress [37]. Thus far, the *CsNPF* genes have not been fully identified. Therefore, in this study, the *NPF* genes in the cucumber genome were identified using bioinformatics methods, and the physicochemical properties, phylogenetic relationship, gene structure, protein motif, and promoter regulatory network of the genes were comprehensively analyzed. In addition, RNA Seq data were analyzed, to study the tissue-specific expression pattern of all *NPF* genes in cucumber, and the expression patterns of *CsNPFs* under different abiotic stress and nitrogen conditions were also investigated, to provide a basis for further exploring the biological function of the cucumber *NPF* gene family.

## 2. Results

### 2.1. Identification of NPF Genes in Cucumber

**Figure 1:**
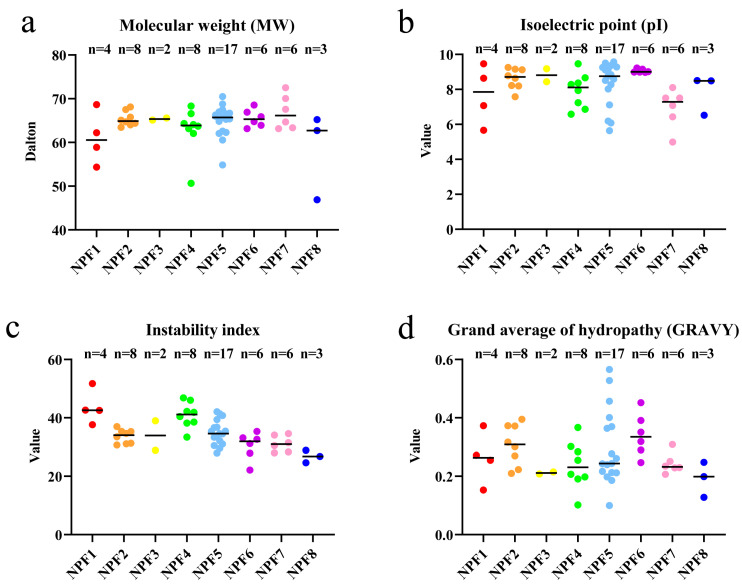
Molecular characterization of NPF proteins in cucumber. (**a**) molecular weight; (**b**) theoretical isoelectric point; (**c**) instability index; (**d**) grand average of hydropathy.

In total, 59 *NPF* genes were identified in cucumber through a BLASTP search against the cucumber genome database (v3.0), according to homology with 53 *AtNPFs* and *OsNPFs* and a hidden Markov model (HMM) search using the NPF domain (PF00854). To demonstrate the reliability of these genes, we searched for NPF domains in 59 protein sequences using CDD (http://www.ncbi.nlm.nih.gov/cdd/ (accessed on 3 August 2022)) and Pfam (http://pfam.xfam.org/search/sequence (accessed on 3 August 2022)). Among these candidates, five sequences with lacking or incomplete domains were removed. Finally, 54 cucumber *NPF* genes were obtained (Table S1). The physicochemical properties of the CsNPF protein sequences were analyzed using ExPASy, as shown in Figure 1 and Table S1. The CsNPF proteins had an average of 579 amino acid residues, ranging from 416 aa (*CsNPF4.7*) to 652 aa (*CsNPF7.4*) (Table S1). The molecular weight (MW) ranged from 46.88 kD (*CsNPF8.1*) to 72.52 kD (*CsNPF7.4*), with a mean value of 64.31 kD and a difference of 25.64 kD between the maximum and minimum values (Figure 1a, Table S1). The isoelectric point (pI) ranged from 4.99 (*CsNPF7.1*) to 9.57 (*CsNPF5.2*), of which nine were acidic proteins (pI less than seven) and 45 were basic proteins (pI higher than seven) (Figure 1b, Table S1). The instability index ranged from 22.14 (*CsNPF6.6*) to 51.72 (*CsNPF1.1*); of these, 11 were greater than 40, indicating unstable proteins (Figure 1c, Table S1). The grand average of hydropathy (GRAVY) ranged from 0.1 (*CsNPF5.7*) to 0.566 (*CsNPF5.12*), indicating that all cucumber NPF family proteins were hydrophobic proteins (Figure 1d, Table S1).

### 2.2. Phylogenetic Analysis of NPF Genes in Cucumber

**Figure 2:**
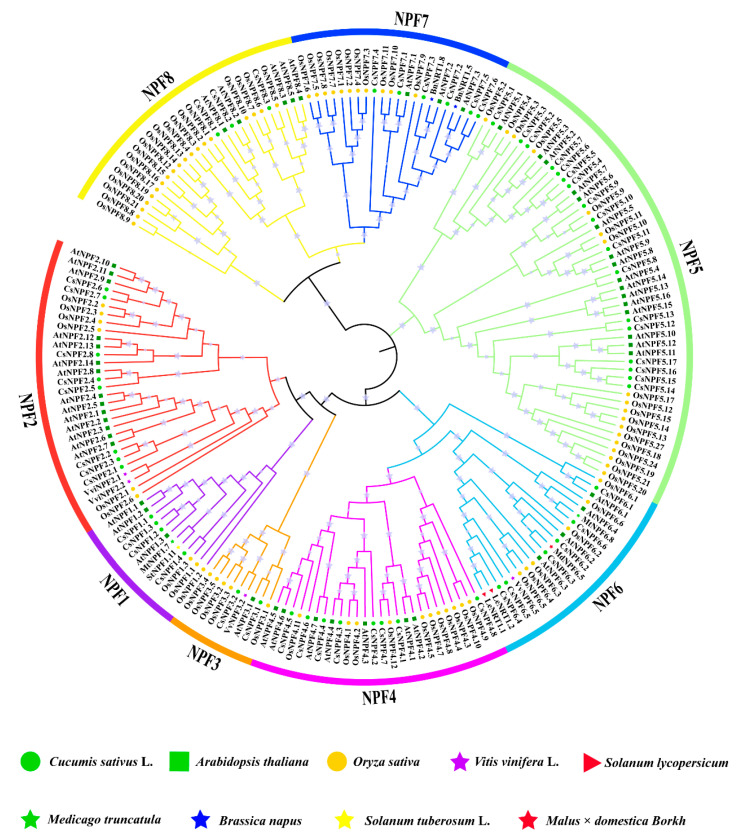
Phylogenetic analysis of the *NPF* gene family. In total, 198 protein sequences, including 54 from cucumber (*Cucumis sativus* L.), 53 from Arabidopsis (*Arabidopsis thaliana*), 79 from rice (*Oryza sativa*), 4 from grapevine (*Vitis vinifera* L.), 2 from tomato (*Solanum lycopersicum*), 2 from barrel medic (*Medicago truncatula*), 2 from rapeseed (*Brassica napus*), 1 from potato (*Solanum tuberosum* L.), and 1 from apple (*Malus × domestica Borkh.*), were involved in the analysis. The clades representing different subfamilies (NPF 1–8) are indicated by different colors.

To classify the cucumber *NPF* genes systematically and reveal their evolutionary relationships with other plant *NPF* genes, multiple sequences of NPFs in different species were aligned using Clustal W, and a phylogenetic tree was constructed by FastTree using a maximum likelihood (ML) analysis. As shown in Figure 2, the cucumber NPF proteins could be categorized into eight subfamilies (NPF1–NPF8), which are similar to those in Arabidopsis and rice. The distribution of CsNPFs in different subfamilies is as follows: NPF1 (4), NPF2 (8), NPF3 (2), NPF4 (8), NPF5 (17), NPF6 (6), NPF7 (6), and NPF8 (3). Among them, NPF1 is more closely related to the NPF2 subfamily on one branch, NPF4 is more closely related to NPF6, and NPF7 is more closely related to NPF8, which is consistent with the results of previous research [8,10,11,38,39]. To distinguish the subfamilies of *NPF* genes, we renamed all *CsNPF* genes according to the international nomenclature, based on their homology with *AtNPF* genes (Table S1) [40].

### 2.3. Gene Structure, Domain, and Conserved Motif Analyses

**Figure 3:**
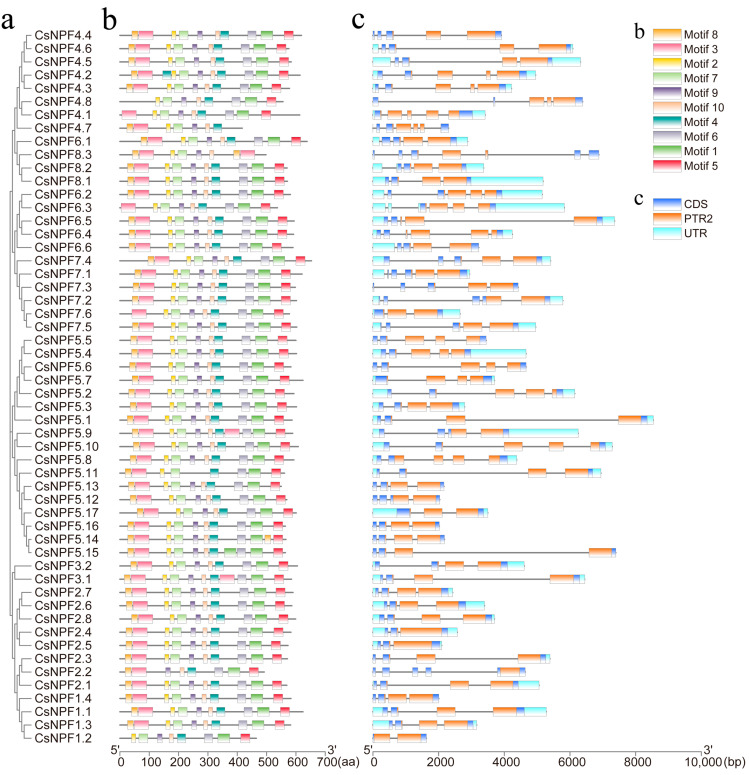
Phylogenetic relationships, conserved protein motifs, and gene structure of *CsNPF* genes. (**a**) Phylogenetic tree of the *CsNPF* gene family. (**b**) Conserved motif analysis of CsNPF protein sequences. Each motif is represented by a specific color. (**c**) Gene structure and domain analyses of *CsNPF* genes. PTR2 domain, CDS and UTR, which are indicated by orange, dark blue, and wathet boxes, respectively.

Exon–intron structural diversity can reveal the evolution of multigene families. To acquire more insights into the gene structure, we analyzed the exon–intron structure of the cucumber NPFs. The results showed that the number of introns in the *CsNPF* genes ranged from one to six (Figure 3c). Most members (22) had four introns, 21 members had three introns, four members had two or five introns, two members had six introns, and *CsNPF1.2* had the lowest number of introns, with only one. Three introns are highly conserved in most *CsNPF* genes in terms of the insertion sites, as follows: one intron inserts in front of the PTR2 domain, and two introns insert into the PTR2 domain. Therefore, these three introns may be essential for the function of *CsNPF* genes [41].

The protein sequence analysis demonstrated that all CsNPFs contained the PTR2 domain. The motif distribution of 54 CsNPF proteins was analyzed using the MEME program, and 10 motifs were identified and named motif1–motif10. As shown in Figure 3b, motifs 2, 4, 7, 9, and 10 are present in 53 CsNPF protein sequences; motifs 1, 3, 5, and 6 are present in 52 CsNPF protein sequences; and motif 8 is present in 49 CsNPF protein sequences. Almost all CsNPF proteins have similar motif compositions, indicating that the protein structure of CsNPF is conserved. Moreover, *CsNPF8.3* does not contain motifs 1, 4, 5, and 6, but contains two motifs, motif 3 and motif 8, that are significantly different from those in other genes and may have a functional differentiation.

### 2.4. Analysis of Cis-Acting Regulatory Elements

**Figure 4:**
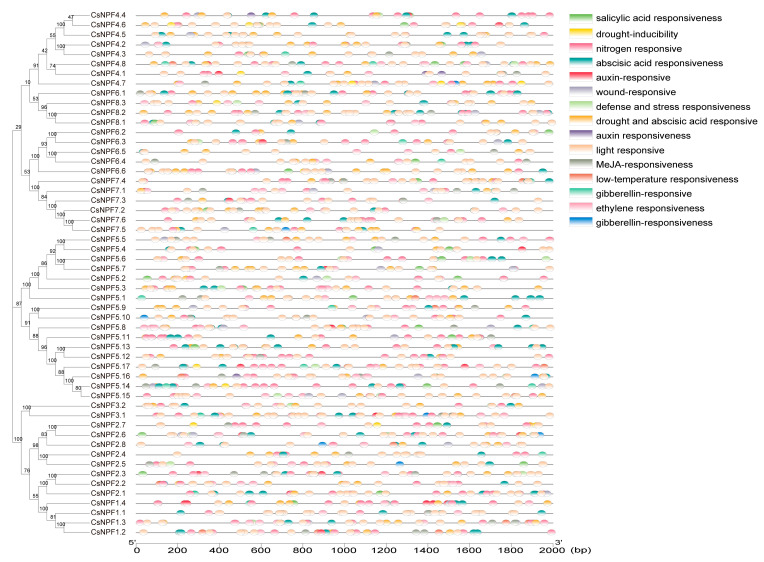
*Cis*-acting regulatory element (CRE) analysis in the 2000 bp promoter regions of the 54 *CsNPF* genes.

The *cis*-acting regulatory elements (CREs) in promoter regions play significant roles in the transcriptional regulation of downstream genes, by binding transcription factors. We isolated the CREs in the promoter regions (−2000 bp) of cucumber NPFs using PlantCARE. The results showed that, among the 54 *CsNPF* genes, in addition to the core elements (CAAT-box and TATA-box), the genes contained a number of elements related to abiotic stresses (MYB, MYC, WUN-motif, TC-rich repeats, LTR, and MBS), hormones (ABRE, ERE, CGTCA-motif, TGACG-motif, TCA-element, TGA-element, P-box, GARE-motif, TATC-box, AuxRR-core, AuxRE, and TGA-box), light response (Box 4, G-Box, and MRE), and cis-acting elements related to tissue organ development (CAT-box and GCN4_motif) (Figure 4, Table S2). It is hypothesized that the expression of *CsNPF* genes may be coregulated by multiple signals, such as light signals, hormones, and stress responses. It has been reported that the GATA motif, MYB, and G box are involved in the molecular regulation of plant nitrogen status [42,43]. Thus, all *NPF* family genes may be associated with the nitrogen response.

### 2.5. Chromosomal Localization and Synteny Analysis of CsNPF Genes

**Figure 5:**
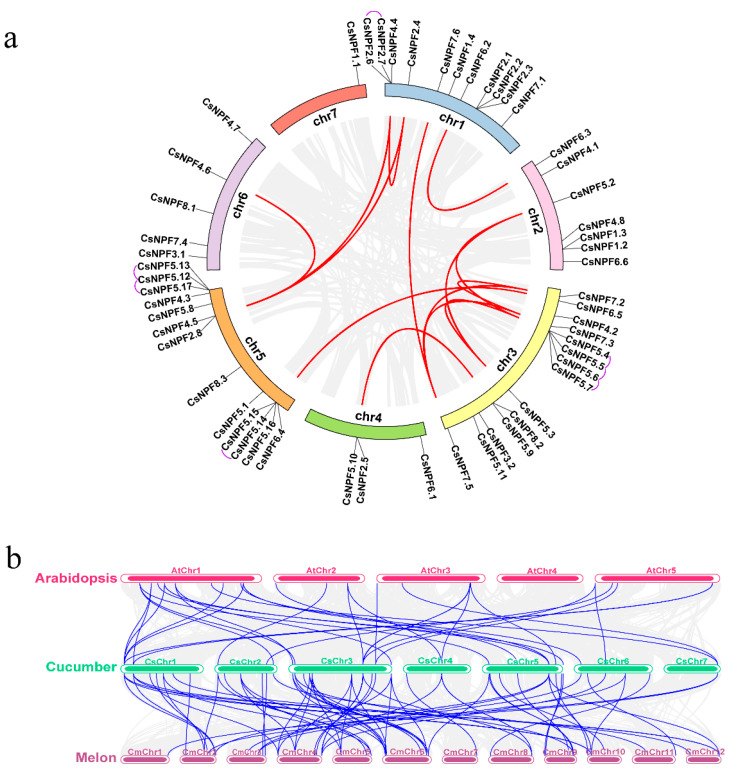
Chromosomal distribution, gene duplication, and synteny analysis of *CsNPF* genes. (**a**) Schematic representations of the chromosomal distribution and interchromosomal relationships of *CsNPF* genes. Gray lines in the background indicate all syntenic blocks in the cucumber genome, red lines indicate segmental duplication gene pairs, and purple lines in the outer ring indicate tandem duplication gene pairs. (**b**) Synteny analysis of *NPF* genes between cucumber and Arabidopsis and melon. The gray lines indicate all collinear blocks, and the blue lines indicate collinear *NPF* gene pairs.

The chromosomal localization analysis revealed that 54 *CsNPF* genes were heterogeneously distributed on seven chromosomes of cucumber (Figure 5a). Chromosome (Chr) 3 contained the largest number of *CsNPF* genes (14 genes), followed by Chr5 (13 genes), and Chr4 (3 genes), while Chr7 had the fewest *NPF* genes (1 gene). The homology analysis of cucumber *NPF* genes using BLASTP and the Multiple Collinearity Scan toolkit (MCScanX) identified seven tandem duplication gene pairs in the *CsNPF* genes (Figure 5a), which were distributed on the same chromosome and had high coding similarity. Fifteen segmental duplication gene pairs were also identified, and these results reflect that the amplification of cucumber *NPF* genes may originate from tandem and segmental replication.

To further explore the phylogenetic mechanisms of the *NPF* gene family in cucumber, we conducted a comparison of the syntenic map of cucumber associated with Arabidopsis and melon. In total, 71 *NPF* collinear gene pairs were identified between cucumber and melon, and 37 gene pairs were identified between cucumber and Arabidopsis (Figure 5b). Both cucumber and melon belong to Cucurbitaceae, 80% of *CsNPFs* showed a syntenic relationship with *NPFs* in melon, and *NPF* genes in cucumber and melon were highly evolutionarily conserved. *CsNPF1.1*, *CsNPF2.4*, *CsNPF2.6*, *CsNPF2.8*, *CsNPF4.4*, *CsNPF4.5*, *CsNPF5.9*, and *CsNPF5.10* correspond to two or more syntenic gene pairs in melon and Arabidopsis, and it is hypothesized that these *CsNPF* genes may play significant roles in the evolution of the *NPF* gene family. In addition, *CsNPF1.3*, *CsNPF3.1*, and *CsNPF5.8* were found to be associated with one syntenic gene pair between cucumber and melon/Arabidopsis, suggesting that these *NPF* genes may have evolved from the same ancient *NPF* genes. However, some *CsNPF* genes do not exist as syntenic gene pairs with Arabidopsis and melon, suggesting that these genes may have been specific to cucumber during its evolution.

### 2.6. Expression Profiles of CsNPF Genes in Different Organs

**Figure 6:**
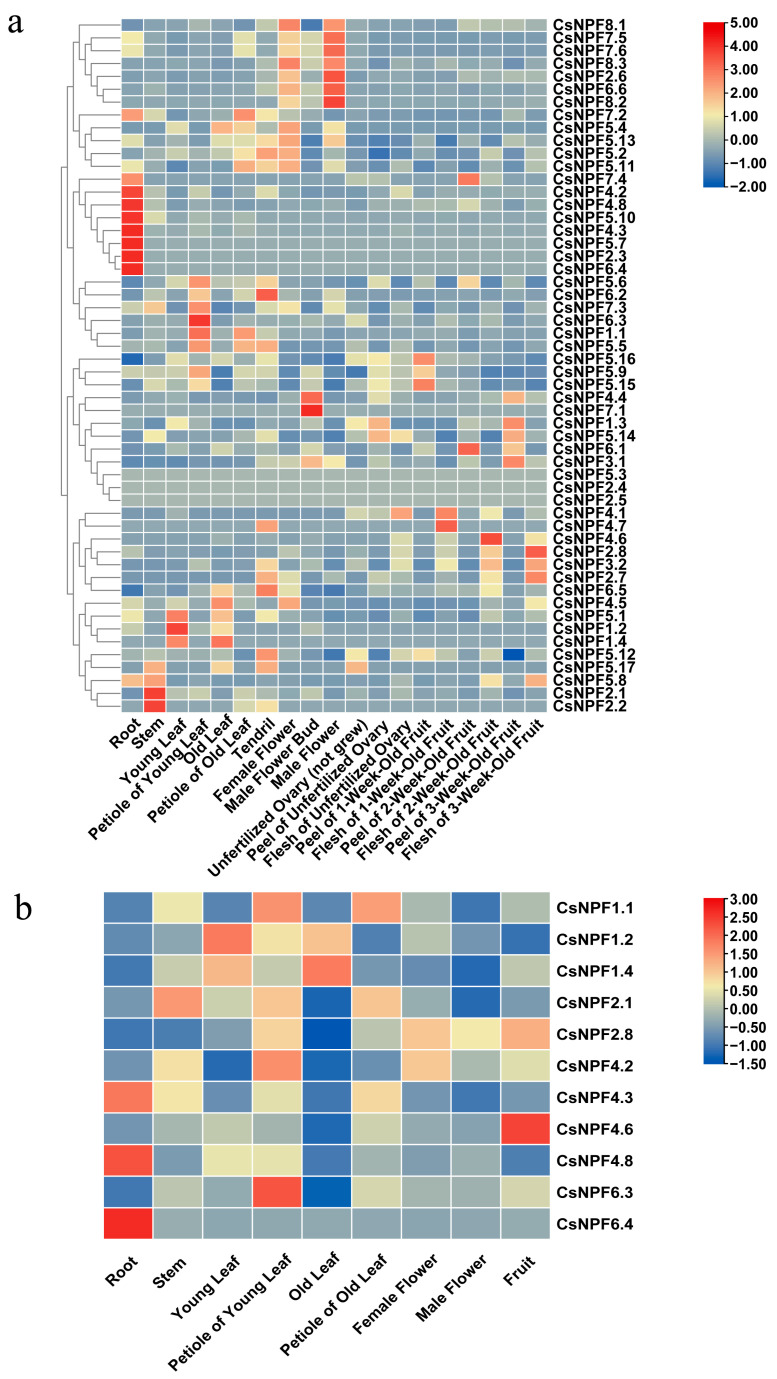
Tissue-specific expression of *NPF* genes in cucumber. (**a**) The expression profiles of *CsNPF* genes in 19 tissues were investigated based on public transcriptome data. Gene expression levels were evaluated as log2−transformed fragments per kilobase of exon model per million mapped fragments (FPKM) values. (**b**) Transcript levels of 12 *CsNPFs* were analyzed using qRT–PCR in roots, stems, young leaves, petiole of young leaves, old leaves, petiole of old leaves, female flowers, male flowers, and fruits. Data from cucumber roots were selected as standards, to measure the relative expression of genes in other tissues, and the expression levels of each gene in roots was set as 1.

Based on the public transcriptome data of 19 different tissues of cucumber, the expression patterns of 54 *CsNPF* genes in different organs were investigated using a standard transfer group analysis program. The results indicate that members of the cucumber *NPF* family showed differences in organ-specific expression patterns (Figure 6a, Table S3). *CsNPF2.3*, *CsNPF5.7*, and *CsNPF6.4* were specifically expressed in roots; and *CsNPF4.2*, *CsNPF4.3*, *CsNPF4.8*, and *CsNPF5.10* were highly expressed in roots; *CsNPF2.1* and *CsNPF2.2* were highly expressed in stems; *CsNPF1.1* was expressed at high levels in young and old petioles; while *CsNPF5.6*, *CsNPF6.3*, and *CsNPF7.3* were only highly expressed in young petioles; *CsNPF7.1* was specifically expressed in the male flower bud; *CsNPF2.6*, *CsNPF6.6*, *CsNPF7.5*, *CsNPF7.6*, *CsNPF8.1*, *CsNPF8.2*, and *CsNPF8.3* exhibited high expression in both female and male flowers; and *CsNPF2.8*, *CsNPF3.2*, and *CsNPF4.6* showed high expression levels during the development of fruits. The analysis of these expression patterns is helpful for understanding the function of *CsNPF* genes in cucumber.

We examined the expression of tissue-specific expressed genes screened from RNA-seq data from the root, stem, young leaf, petiole of young leaf, old leaf, petiole of old leaf, female flower, male flower, and fruit using qRT–PCR. The expression of selected genes was consistent with the RNA-seq data, except for *CsNPF4.2*, which had a low expression in the roots and high expression in the petioles of young leaves (Figure 6b).

### 2.7. Expression Profiles of CsNPF Genes under Abiotic Stresses

**Figure 7:**
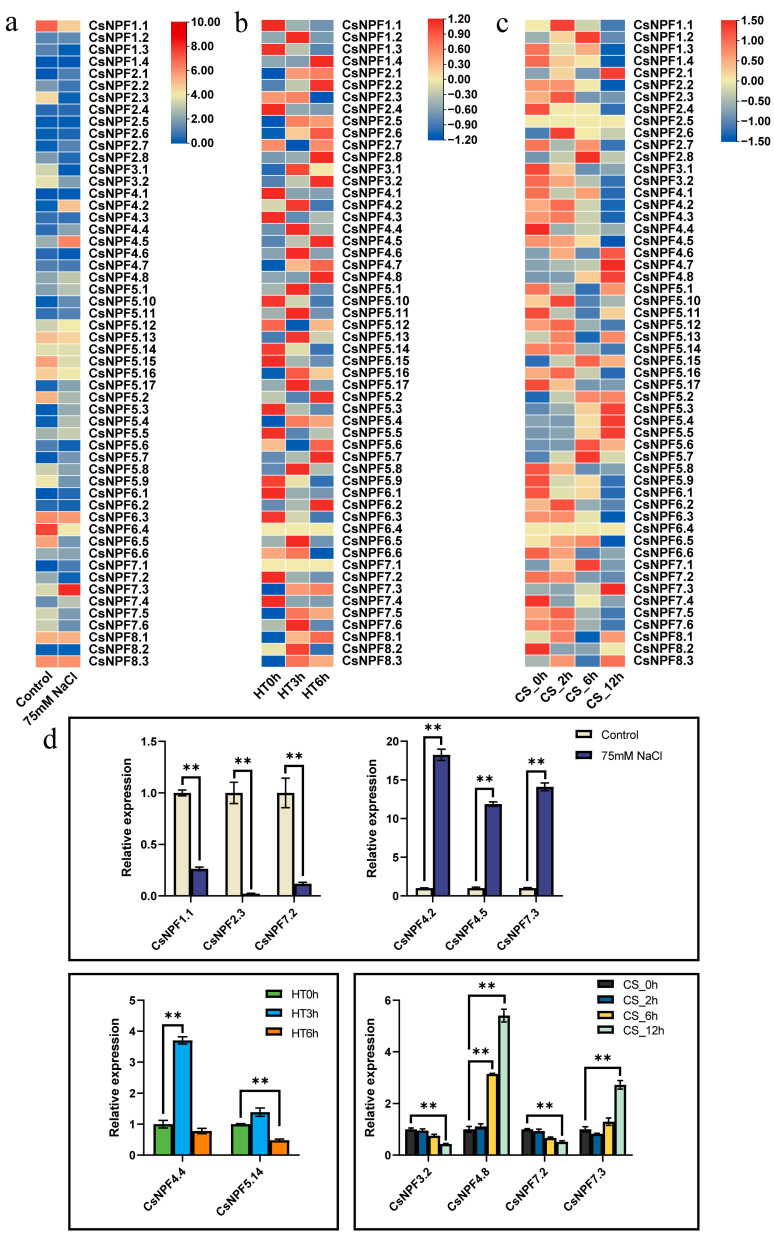
Expression profiles of *CsNPF* genes in response to various abiotic stress treatments. (**a**) Salt stress. (**b**) Heat stress. (**c**) Cold stress. Gene expression levels were evaluated as log2−transformed FPKM values. CK, control; 75 mM NaCl, 75 mM NaCl treatment after 24 h; HT0h, heat treatment for 0 h; HT3h, heat treatment for 3 h; HT6h, heat treatment for 6 h; CS_0h, untreated; CS_2h, exposed to an air temperature of 6 °C for 2 h; CS_6h, exposed to an air temperature of 6 °C for 6 h; CS_12 h, exposed to an air temperature of 6 °C for 12 h; (**d**) Relative expression levels of 10 *CsNPF* genes under salt, heat, and cold stresses using qRT–PCR. A significant difference between the different treatment was indicated by Tukey’s method (** *p* < 0.01). Data from control, HT0h, and CS_0h were selected as standards to measure the relative expression of genes under salt stress, heat stress, and cold stress, respectively, and the expression level of each gene under the control, HT0h, and CS_0h treatments was set to 1.

We analyzed the response of *CsNPFs* to different abiotic stress treatments based on public transcriptome information. The results showed that 12 genes were significantly upregulated and 10 genes were significantly downregulated under salt stress (Figure 7a, Table S4). Moreover, we analyzed the expression of *CsNPFs* in cucumber leaves after heat and cold treatment at different time points. *CsNPF4.4* was significantly induced after 3 h of heat treatment, and *CsNPF5.14* was significantly inhibited after 6 h of heat treatment (Figure 7b, Table S4). Under cold stress, the differential expression of *CsNPFs* mainly occurred 12 h after treatment. We discovered that the expression levels of *CsNPF4.8* and *CsNPF7.3* were significantly increased in response to cold stress (Figure 7c, Table S4). In addition, nine genes (*CsNPF3.2*, *CsNPF4.1*, *CsNPF4.2*, *CsNPF4.3*, *CsNPF5.9*, *CsNPF6.2*, *CsNPF6.3*, *CsNPF7.2* and *CsNPF7.5*) showed opposite trends following exposure to cold stress.

We further examined the expression patterns of 10 *CsNPF* genes under salt, heat, and cold stresses using qRT–PCR. We found that the expression of selected genes was consistent with the RNA-Seq data (Figure 7d).

### 2.8. Expression Profiles of CsNPF Genes under Different Nitrate Concentrations

**Figure 8:**
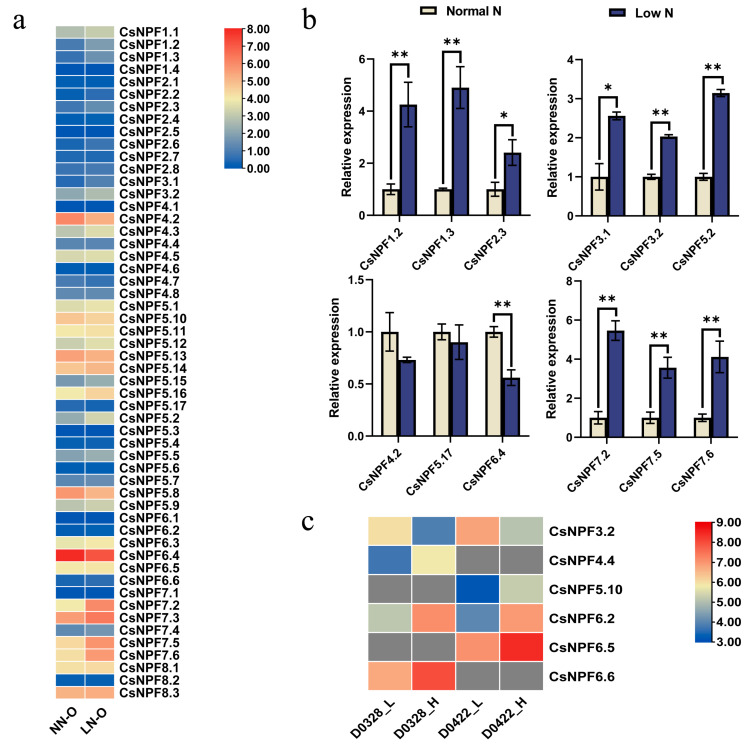
Expression profiles of *CsNPF* genes under different N treatments. (**a**) The expression patterns of 54 *CsNPF* genes under N deficiency treatments in cucumber roots. The expression data were obtained from RNA-seq data and are shown as log2−transformed FPKM values. NN-O, normal nitrogen treatments; LN-O, N deficiency treatments. (**b**) Relative expression levels of 12 *CsNPF* genes under N deficiency treatments using qRT–PCR in roots. A significant difference between the different treatments was indicated by Tukey’s method (* *p* < 0.05, ** *p* < 0.01). Data from cucumber roots treated with normal nitrogen were selected as standards, to measure the relative expression of genes in other nitrogen treatments, and the expression levels of each gene under normal nitrogen treatment was set as 1. (**c**) Differentially expressed *CsNPF* genes identified from D0328 and D0422 plants grown under low and high N conditions. D0328_L, D0328 grown under low N conditions; D0328_H, D0328 grown under high N conditions; D0422_L, D0422 grown under low N conditions; D0422_H, D0422 grown under high N conditions.

To investigate the response of *CsNPFs* to different nitrogen supplies in cucumber roots, we performed an expression analysis of *CsNPFs* after nitrogen stress treatment based on RNA-Seq data. The expression profiles of the *CsNPF* genes were significantly altered under N deficiency conditions. Compared with the nitrogen-sufficient condition, 12 *CsNPF* (*CsNPF1.2*, *CsNPF1.3*, *CsNPF2.3*, *CsNPF2.6*, *CsNPF3.1*, *CsNPF3.2*, *CsNPF5.2*, *CsNPF5.15*, *CsNPF7.2*, *CsNPF7.3*, *CsNPF7.5* and *CsNPF7.6*) genes were significantly upregulated by N deficiency, and three genes (*CsNPF4.2*, *CsNPF5.8* and *CsNPF6.4*) were significantly downregulated (Figure 8a, Table S5).

To explore the roles of *NPF* genes in response to nitrate, we further confirmed the expression patterns of 12 *CsNPF* genes under N deficiency treatments in cucumber roots using qRT–PCR. We found that the expression of the selected genes was consistent with the RNA-Seq data (Figure 8b).

An expression analysis of *CsNPF* genes was performed based on transcriptome data from the leaves of two cucumber cultivars with high (D0328) and low (D0422) capacities for low N tolerance under different N nutrient supply conditions [44]. Four differentially expressed *CsNPF* genes were identified in each of the two cultivars (Figure 8c). Under low nitrogen conditions, *CsNPF6.2* was significantly downregulated, and *CsNPF3.2* was significantly upregulated in both cucumber cultivars. Notably, *CsNPF3.2* was significantly upregulated in cucumber roots and leaves under low nitrogen, indicating that it may play an important role in low nitrogen stress tolerance in cucumber.

## 3. Discussion

*NPF* genes are involved in the uptake and transport of nitrate in plants, are essential for plant growth and development, and have been reported in many species. However, the number of *NPF* family members varies to a large extent among species. In this study, we identified 54 *NPF* genes in the whole genome of cucumber and performed a phylogenetic analysis. There are two classification systems for the *NPF* gene family, as follows: one system is based on a phylogenetic analysis of 1077 sequences from 22 plant genomes and 24 nonplant sequences, dividing the family into 10 supergroups and 32 groups [45]; and the other system divides the NRT1/PTR family into eight subfamilies (NPF1-NPF8), based on an analysis of 2398 sequences from 33 full-sequence genomes [40]. Previous studies have generally conformed to the criteria of the latter division [8,11,38,46,47,48,49,50,51,52]. Recently, a new classification method appeared in *Brassica napus*. Three of the eight subfamilies (NPF2, NPF5, and NPF6) were further divided into two subfamilies with higher bootstrap values [41]. Our classification was consistent with the second traditional classification system, and the *CsNPF* genes were divided into eight subfamilies. To confirm the reliability of this result, a phylogenetic analysis of *NPF* genes in several species, such as Arabidopsis, rice, and cucumber, was carried out, and the results supported our classification.

Tandem and segmental duplications play essential roles in the expansion and evolution of gene families. Fourteen segmental duplication events and seven tandem duplication gene pairs were found in the *CsNPF* gene family. A comparison of NPF members in different species shows that there is gene expansion and deletion in the *CsNPF* gene family. For instance, the branch in clade NPF2 had 14 *AtNPF* genes but eight *CsNPF* genes, and NPF8 had five *AtNPF* genes but three *CsNPF* genes. In contrast, the branch in clade NPF7 had three *AtNPF* genes but six *CsNPF* genes, and NPF3 had one *AtNPF* gene but two *CsNPF* genes. *AtNPF6.2* is the ortholog of two cucumber proteins (*CsNPF6.2* and *CsNPF6.3*), while *AtNPF2.12*, *AtNPF2.13*, and *AtNPF2.14* are the orthologs of only one cucumber gene (*CsNPF2.8*). In general, the gene function of a branch is highly conserved in different plant species, but the occurrence of replication and deletion events between genes leads to complexity and functional differences among *NPF* genes in different plant genomes. To accurately identify the true homologous genes among plant species, we performed a synteny analysis of cucumber, melon, and Arabidopsis. The results showed that the cucumber genome was extensively homologous to the Arabidopsis and melon genomes. Many *CsNPF* genes showed linear relationships with melon and Arabidopsis genes. Cucumber and melon are close relatives, and both belong to Cucurbitaceae. *NPF* genes in cucumber and melon are highly evolutionarily conserved.

To study the regulatory mode of *CsNPF* genes under different conditions, we analyzed the cis-acting elements at the promoter region. Many cis-elements involved in phytohormone responses, such as ABA- (51/54 gene), ethylene- (42/54 gene), MeJA- (37/54 gene), SA- (29/54 gene), GA- (27/54 gene), and IAA- responsive CRE (19/54 gene) (Table S2), were discovered in the promoter region of *CsNPF* genes, indicating their potential hormone induction characteristics. In addition, the promoter of *CsNPF* genes also contains cis-elements responding to different stresses, such as low temperature (LTR), drought (MBS), anaerobic (ARE), and wound (WUN-Motif) stresses. These findings indicate that *CsNPF* genes may play a key role in the various stress responses of cucumber. Therefore, we further analyzed the expression pattern of *CsNPF* genes under different abiotic stresses. *AtNRT1.5* and *AtNRT1.8* showed opposite expression patterns under different environmental stresses [53]. *AtNRT1.8* was significantly upregulated and *AtNRT1.5* was significantly downregulated after Cd^2+^, osmotic, cold, methyl jasmonate, salt, and AgNO_3_ stresses [22]. *CsNPF7.3* and *CsNPF7.2* in cucumber were homologous to *AtNRT1.8* and *AtNRT1.5*, respectively. *CsNPF7.3* and *CsNPF7.2* contain cis-acting elements, such as MYB, MYC, ARE, AS-1, and STRE, in response to stresses. Under low temperature and salt stress, *CsNPF7.3* was significantly induced, and *CsNPF7.2* was significantly inhibited. Similarly to Arabidopsis, the coordination of the opposite regulation by *CsNPF7.2* and *CsNPF7.3* may be a universal mechanism enabling plants to cope with environmental stresses. *AtNPF2.3* is expressed in root pericycle cells and contributes to nitrate translocation to the shoots under salinity [17,53]. *CsNPF2.3* was closely related to *AtNPF2.3* and was specifically expressed in cucumber roots. Differently from *AtNPF2.3*, the expression of *CsNPF2.3* was significantly inhibited under salt stress. Therefore, further experiments are needed to verify whether *CsNPF2.3* can promote the distribution of nitrogen to the shoots under salt stress.

*NPF* genes play an important role in nitrogen utilization, and the functions of different genes vary. The biological functions of more than half (31/53) of the Arabidopsis *NPF* family members have been elucidated, and the functions of *NPF* genes in rice, a food crop, have been relatively well reported. However, the function of *NPF* genes in cucumber has been poorly studied.

*CsNPF1.1* is homologous to *AtNPF1.1* and *AtNPF1.2*. In *Arabidopsis thaliana*, *AtNPF1.1* and *AtNPF1.2* are highly expressed in expanded leaves, participate in the transfer of nitrate from xylem to phloem, and are responsible for redistributing nitrate to developing leaves [25]. However, *CsNPF1.1* shows different expression patterns, is mainly expressed in old and young petioles, and may be involved in the storage of nitrate in petioles. Thus, the protein encoded by *CsNPF1.1* has a homologous protein with *Arabidopsis thaliana* but different physiological functions.

*CsNPF2.8* is homologous to *AtNPF2.12*, *AtNPF2.13*, and *AtNPF2.14*. The amino acid sequences of *AtNPF2.12* and *AtNPF2.13* are highly similar, their phylogenetic relationships are close, but their functions are different [54]. *AtNPF2.12* is engaged in the transport of nitrate to developing seeds and regulates the seed abortion rate under nitrogen starvation, which is only expressed in reproductive tissues, while *AtNPF2.13* is responsible for loading excessive nitrate stored in source leaves into the phloem and promoting nitrate transport from old leaves to new leaves [26,28,53]. *CsNPF2.8* is highly expressed in fruit and may play a role in nitrogen storage of embryos and nitrogen starvation response, which is more similar to the physiological function of *AtNPF2.12*.

*AtNPF6.2/NRT1.4* is a nitrate transporter expressed specifically in the petiole and midvein, which mediates the storage of nitrate in the petiole/midvein and is particularly important for maintaining nitrate balance and leaf development [23,24]. Two homologous genes in cucumber, *CsNPF6.2* and *CsNPF6.3*, are highly expressed in the petioles of young leaves and may be responsible for the storage of nitrate in the petioles of young leaves. Liu et al. showed that low temperature treatment significantly inhibited the expression of *CsNPF6.3* (*CsNRT1.4a*) in petiole and midrib, which may be closely related to nitrate transport in petiole and midrib, which further verified the function of *CsNPF6.3* in petiole [55]. In addition, *CsNPF6.2* was significantly expressed in cucumber tendrils, indicating that it may differentiate into other functions, in addition to mediating nitrate storage.

*CsNPF6.4* and *CsNPF6.5* are clustered together with *LeNRT1.1* and *LeNRT1.2* in tomato; *VvNPF6.5* in grapevine; *AtNPF6.3* in Arabidopsis; and *OsNPF6.3*, *OsNPF6.4*, and *OsNPF6.5* in rice, which are homologous to each other. Among them, *AtNPF6.3* (*NRT1.1*) participates in nitrate absorption from roots and nitrate transport from roots to shoots and acts as a nitrate transporter to regulate many molecular, physiological, and morphological responses to nitrate [13,14]. The two rice orthologous genes *OsNPF6.5* and *OsNPF6.3* of *AtNPF6.3* are different in their N response and utilization, but both participate in the absorption of nitrate in roots and show the potential to improve plant nitrogen use efficiency and yields of rice [56]. *VvNPF6.5* encodes a pH-dependent, dual-affinity nitrate transporter that can regulate nitrate absorption and distribution in grapevines, significantly improve plant nitrogen use efficiency, and participate in the primary nitrate response [57]. *LeNRT1.1* and *LeNRT1.2* mediate the nitrate uptake in tomato [58]. Similarly to *AtNPF6.3*, *CsNPF6.4* is highly expressed in roots and is significantly regulated by N stress in roots (Figure 8a, Table S3), suggesting its important role in N absorption. In addition, *AtNPF6.3* participates in a variety of abiotic stress responses, such as Cd^2+^, H^+^, Na^+^, Pb^2+^ and Zn^2+^, and has a function for drought tolerance in the presence of nitrate. The potential function of *CsNPF6.4* in abiotic stress needs to be explored [53]. However, *CsNPF6.5* was preferentially expressed in tendril but less expressed in roots. The expansion of the number of genes and different expression profiles of *AtNPF6.3* homologs with *Arabidopsis thaliana* showed the differentiation of *AtNPF6.3* homologs in cucumber.

*AtNPF7.3/AtNRT1.5* and *AtNPF7.2/AtNRT1.8* have similar genetic relationships and jointly regulate the long-distance transport of nitrate. *AtNRT1.5* is preferentially expressed in the roots, mediates the outflow of nitrate from root cells, and is loaded into the xylem and transported to the shoot [59]. *AtNRT1.8* is mainly expressed in xylem parenchymal cells and is responsible for unloading nitrate from the xylem [22]. *BnNRT1.5* and *BnNRT1.8* of *B. napus* are highly homologous to *AtNRT1.5* and *AtNRT1.8*, respectively, and these two genes are mainly expressed in the roots of both species and play similar roles in the long-distance transport of nitrate between the roots and shoots [60]. *CsNPF7.3* is homologous to *AtNPF7.2* and *BnNRT1.8*, and *CsNPF7.2* is homologous to *AtNPF7.3* and *BnNRT1.5*. Among them, *CsNPF7.2* was highly expressed in roots and may have physiological functions similar to those of *AtNPF7.3* and *BnNRT1.5*. Some studies have shown that *CsNPF7.2* is upregulated after nitrogen starvation treatment and performs a function in meeting the high requirement of N in the vascular xylem and cambium [37]. In our study, the expression trend of *CsNPF7.2* in cucumber roots under nitrogen starvation was consistent with previous studies, suggesting that it may regulate the growth of cucumber seedlings under nitrogen stress. *CsNPF7.3* was highly expressed in stems and at the petioles of young leaves but not significantly expressed in roots and may not have the function of root unloading of NO_3_^−^ in roots.

*CsNPF6.6*, *CsNPF7.1*, and *CsNPF8.2* were significantly expressed in floral organs. *CsNPF6.6* and *CsNPF7.1* are homologous to *AtNPF6.4* and *AtNPF7.1* in Arabidopsis, respectively. These two genes in cucumber may have different functions from their homologous genes in Arabidopsis. *AtNPF6.4* is involved in polyamine tolerance, and *AtNPF7.1* affects nitrogen output in leaves through source–sink regulation and participates in nitrogen cycling under low nitrogen stress. *CsNPF6.6* and *CsNPF7.1* may play important roles in the development of floral organs. *CsNPF8.2* is mutually homologous to *AtNPF8.1* and *AtNPF8.2*. *AtNPF8.1* encodes a dipeptide and tripeptide transporter protein that recognizes a wide range of amino acid combinations and is involved in the absorption of oligopeptides in roots [61]. The dipeptide transporter encoded by *AtNPF8.2* is expressed in pollen and ovules during early seed development and transports oligopeptides to reproductive organs [62]. *CsNPF8.2* may play a role in the transport of oligopeptides to flower organs.

Some genes in the cucumber *NPF* family are specifically expressed in certain organs, but their function has not been determined. For example, *CsNPF4.2*, *CsNPF4.3*, and *CsNPF4.8* in the fourth subfamily of the *NPF* family are highly expressed in roots and are homologous to *AtNPF4.3*, *AtNPF4.4*, and *AtNPF4.12* in Arabidopsis, respectively. *CsNPF2.1* and *CsNPF2.2* are specifically expressed in the stem and may be involved in the transport of NO_3_^−^ in the stem. *CsNPF1.2* and *CsNPF1.4* are highly expressed in cucumber leaves, and their homologous genes in model plants are *AtNPF1.3* and *OsNPF1.3.* To date, the function of these homologous genes in Arabidopsis and other plants has not been reported. Thus, the functions of the whole *NPF* gene family in the higher plants are far from being clarified and require further exploration.

## 4. Materials and Methods

### 4.1. Genome-Wide Identification and Characterization of CsNPF Genes

The whole genome sequences of cucumber were obtained from the Cucurbit Genomics Database (http://cucurbitgenomics.org/ftp/genome/cucumber/Chinese_long/v3/ (accessed on 1 August 2022)). The full-length sequences of Arabidopsis and rice *NPF* genes were retrieved from the Arabidopsis genome database (https://www.arabidopsis.org (accessed on 1 August 2022)) and Rice Genome Annotation Project (http://rice.uga.edu/index.shtml (accessed on 1 August 2022)), respectively. First, a two-step BLAST method was used to identify cucumber *NPF* genes. The Arabidopsis and rice NPFs were used as queries for the BLAST analysis in TBtools (e-value, 1 × 10^−10^), and then the obtained protein sequences were further identified using National Center for Biotechnology Information (https://www.ncbi.nlm.nih.gov/ (accessed on 1 August 2022)) BLASTP (e-value, 1 × 10^−10^), to avoid false-positives. Second, the HMM profiles of the NPF domain (PF00854) were obtained from the Pfam database (http://pfam.xfam.org/ (accessed on 1 August 2022)) [63], and the *NPF* genes in the Cucumber Genome Database (v3.0) were identified by HMMER 3.0. The results of the two parts were merged, and duplicate sequences were removed. The preliminary sequences were then validated using CDD (http://www.ncbi.nlm.nih.gov/cdd/ (accessed on 3 August 2022)) [64] and Pfam (http://pfam.xfam.org/search/sequence (accessed on 3 August 2022)) [63] databases. Candidate sequences lacking, or with incomplete, domains were removed. The MW, PI, instability index, and GRAVY values of the candidates were calculated using ExPASy (https://www.expasy.org/resources/protparam (accessed on 5 August 2022)) [65].

### 4.2. Sequence Alignment and Phylogenetic Analysis

The *NPF* genes of Arabidopsis and rice were used for a phylogenetic analysis along with the *NPF* gene of cucumber. Multiple sequence alignment of candidate protein sequences was performed using the Clustal W [66] program with the default parameters. A phylogenetic tree was constructed using FastTree [67] with the ML method based on the LG model, and a bootstrap test was carried out with 1000 iterations. The trees were visualized and refined using Interactive Tree of Life (ITOL, https://itol.embl.de/itol.cgi (accessed on 1 September 2022)) [68].

### 4.3. Gene Structure, Domain, and Conserved Motif Analyses

The introns and exons of each *CsNPF* gene were identified using TBtools [69]. The conserved motifs of cucumber NPFs were analyzed using MEME (https://meme-suite.org/meme/tools/meme (accessed on 6 September 2022)) [70] with the default parameters, except that the maximum number of motifs was set to 10. Batch-CD Search with the default parameters was used to analyze the conserved domains. All results were integrated and visualized using TBtools.

### 4.4. Cis-Regulatory Element Analysis of CsNPF Genes

The 2000 bp upstream of the initiation codon of each *CsNPF* gene was extracted using Tbtools. The cis-regulatory elements were identified using PlantCARE (http://bioinformatics.psb.ugent.be/webtools/plantcare/html/ (accessed on 15 September 2022)) [71].

### 4.5. Chromosomal Location and Collinearity Synteny Analysis

The chromosomal location information was acquired from the gff3 files of the cucumber genome and visualized using TBtools. The MCScanX [72] was analyzed to identify gene duplication events and collinearity relationships with the default parameters. To explore the collinearity relationships of the orthologous *NPF* genes in cucumber with those in other selected species, collinearity maps were constructed using MCScanX and visualized using the multiple synteny plot tool in TBtools.

### 4.6. RNA-seq Data Analysis of CsNPF Genes

To analyze the expression patterns of *CsNPF* genes, RNA-seq data from BioProject PRJNA312872 of the Cucurbit Expression Atlas were explored [73]. Clean tags were relocated to the cucumber genome sequence (http://cucurbitgenomics.org/ (accessed on 8 October 2022), v3.0) using the Transcriptome Data Analysis plugin in TBtools, and the FPKM values were recalculated. Twenty-three different tissues of cucumber, including roots, stems, young leaves, young petioles, old leaves, old petioles, tendrils, female flowers, male flowers, ovaries, expanded unfertilized ovaries, and expanded fertilized ovaries, were analyzed.

The expression profiles of *CsNPF* genes in response to different stresses were obtained from publicly available transcriptomic data, and the differentially expressed genes were analyzed after treatment with salt (GSE116265) [74], heat (GSE151055) [75], and cold (GSE111998). The expression levels of *CsNPF* genes are shown on a heatmap using TBtools.

### 4.7. Plant Material and Treatment

Cucumber (*Cucumis sativus* L. “Jinchun 4”) seeds were sterilized in 55 °C hot water for 20 min and germinated on moist filter paper in a dark incubator at 28 °C for 24 h. Then, the germinated seeds were sown in hydroponic seedling plates with distilled water in a light incubator for growth (relative humidity: 70%, 14 h/10 h light/dark; 25 °C/18 °C day/night). When the cotyledons started spreading, the seedlings were watered with modified 1/2 Hoagland nutrient solution. Once the first true leaves appeared, the seedlings were transferred to hydroponic boxes filled with modified Hoagland solution (pH 6.0–6.5) containing the following: 4 mM Ca (NO_3_)_2_, 3 mM KNO_3_, 1 mM NH_4_H_2_PO_4_, 2 mM MgSO_4_·7H_2_O, 3 mM KCl, 71.24 μM EDTAFeNa_2_, 46.24 μM H_3_BO_3_, 9.55 μM MnSO_4_·H_2_O, 0.77 μM ZnSO_4_·7H_2_O, 0.32 μM CuSO_4_·5H_2_O, and 0.5 µM Na_2_MoO_4_·2H_2_O. The nutrient solutions were renewed every 2 d, and air was supplied intermittently to the cassette. When the cucumber seedlings grew to the four-leaf stage, some seedlings were transferred to nitrogen-free nutrient solution (0 mM NO_3_^−^, 0 mM NH_4_^+^) for nitrogen deficiency treatment, and the others were kept under nitrogen-sufficient conditions as controls. After 2 h, roots were sampled from three independent replicates, immediately frozen in liquid nitrogen, and stored at −80 °C.

All samples were subjected to transcriptome sequencing by BioMarker (Beijing, China). The transcript abundance of *CsNPF* genes was calculated as FPKM.

### 4.8. Quantitative Real-Time PCR Analysis

We screened 11 genes with tissue-specific expression in the tissue expression profile and 12 differentially expressed genes in the transcriptome data treated with nitrogen deficiency for the qRT–PCR analysis. Primers were designed using Primer Premier 5 (Table S6). An RNAiso Plus kit (TaKaRa, Dalian, China) was used to isolate the total RNA from various tissues of cucumber and samples of nitrogen-treated roots. cDNA was synthesized from 1 μg of total RNA using a PrimeScript™ RT Reagent Kit with gDNA Eraser (TaKaRa, Dalian, China). A real-time PCR analysis (qRT–PCR) was performed using a One Step SYBR PrimeScript RT–PCR kit (TaKaRa, Dalian, China) with a CFX96 Touch™ Real-Time PCR Detection System (Bio-Rad, Berkeley, CA, USA). The cycling protocol was as follows: 3 min at 95 °C, 40 cycles of 15 s at 95 °C, 30 s at 55 °C, and 15 s at 72 °C. Normalization was performed using the 18S rRNA gene (Csa2G252100). The 2^−∆∆Ct^ method was applied to calculate the relative expression level [76].

### 4.9. Statistical Analysis

The data are presented as the means and standard deviations of three observations. The data were analyzed using SPSS 22 software (IBM, Chicago, IL, USA). Tukey’s *t* test was used to assess the significant difference at a 5% level.

## 5. Conclusions

In this study, we identified 54 *CsNPF* genes in the cucumber genome and categorized them into eight subfamilies, each of which has a conserved structure. Through RNA-Seq and qRT–PCR analyses, we found that the expression patterns of *CsNPF* genes varied with the tissue type, nitrate supply in the nutrient solution, and abiotic stress, indicating that they had diverse functions in nitrate utilization and response to abiotic stress. Among which, *CsNPF6.4* may participate in the process of nitrate absorption and transport in cucumber roots, *CsNPF6.3* may be responsible for NO_3_^−^ storage in petioles, and *CsNPF2.8* may play an important role in NO_3_^−^ transport to embryos. In addition, *CsNPF7.3* and *CsNPF7.2* have significant differences under salt, cold, and low nitrogen stress, which may play an important role in plant adaptation to environmental stress. Our results provide fundamental information for further studies investigating the specific functions of individual genes in the cucumber *NPF* gene family and lay a foundation for manipulating these *NPF* genes, to improve cucumber nitrogen use efficiency and ultimately reduce nitrogen use in cucumber production.

## Data Availability

Data are contained within the article or Supplementary Materials.

## Supplementary Materials

The following supporting information can be downloaded at: https://www.mdpi.com/article/10.3390/plants12061252/s1, Table S1: Characteristics of the NPF family genes in cucumber. Table S2: The cis-regulatory elements identified in the promoter region of CsNPF genes. Table S3: Expression profiles of CsNPF genes in different organs. Table S4: RNA-seq of CsNPF genes under salt, heat, and cold stress. Table S5: FPKM values of CsNPF genes under N deficiency treatments. Table S6: List of qRT–PCR primers used in this study.
